# Computationally Probing the Role of Time‐Limited Neuronal Plasticity in Early Visual Development

**DOI:** 10.1111/desc.70099

**Published:** 2025-11-22

**Authors:** Marin Vogelsang, Lukas Vogelsang, Pawan Sinha

**Affiliations:** ^1^ Department of Brain and Cognitive Sciences MIT Cambridge Massachusetts USA

**Keywords:** acuity, adaptive initial degradations, color, deep neural networks, perceptual development, visual development

## Abstract

In the first months or years of life, newborns learn to perceive their environment at a remarkable pace. This capacity is often attributed to heightened neuronal plasticity that diminishes over time. While this decline in plasticity could be seen as constituting a mere biological constraint, we ask whether it might, in fact, serve an adaptive function. A natural consequence of time‐limited plasticity is the preservation of sensory processing mechanisms formed early in life. Emerging evidence has supported the “adaptive initial degradation” hypothesis, which posits that early experience with degraded sensory inputs, such as blurred or color‐reduced vision, helps instantiate important sensory mechanisms subserving robust perception later in life. Here, we employ deep neural networks as computational models to systematically probe how developmental improvements in visual fidelity interact with diminishing plasticity, modeled via decreasing learning rates. Across both acuity and color domains, we find that time‐limited plasticity yields modest performance gains and helps stabilize early‐formed representations. However, these improvements remain surprisingly modest. Further analysis of the networks’ internal representations also reveals that earlier layers stabilize sooner than deeper layers, loosely paralleling findings from biological development. Overall, our results suggest that while declining plasticity can confer small benefits, it is not the primary force behind the more pronounced advantages of commencing visual experience with degraded inputs. These insights refine the “adaptive initial degradation” hypothesis and, more broadly, underscore how computational modeling can shed light on longstanding questions in developmental neuroscience.

**Summary:**

- Time‐limited neuronal plasticity is often viewed as a mere biological constraint. However, might it provide adaptive advantages by preserving sensory mechanisms learned early in development?
- Simulations with initially degraded sensory inputs reveal modest performance gains when modeling time‐limited plasticity as decreasing learning rates.
- Despite these gains, plasticity reductions contribute less than the benefits conferred by starting with degraded inputs, refining the “adaptive initial degradation” hypothesis.
- These results underscore the utility of computational models to illuminate questions in developmental neuroscience and point to interesting future research avenues.

## 1 Introduction

A hallmark of human development, and that of many other animals, is the dramatically high learning capacity observed early in life, followed by a gradual decrease with age. A classic illustration is how children can effortlessly acquire a new language and sound like native speakers. In contrast, adults who begin later typically retain accents or face grammatical challenges. Such observations, documented extensively in past empirical studies (Johnson and Newport 1989; Hartshorne et al. 2018), have been conceptualized as demonstrating the existence of “critical” or “sensitive” periods, which refer to limited developmental windows of heightened plasticity early in life (Power and Schlaggar 2017). These periods are not restricted to the above example of language acquisition but also apply to sensory systems, notably vision. From the foundational deprivation studies of Hubel and Wiesel (Wiesel and Hubel 1965) to investigations of children gaining sight after months (Lewis and Maurer 2005) or even years (Ostrovsky et al. 2006) of blindness, as well as studies of amblyopia (Hensch and Quinlan 2018), previous research has supported the notion of critical periods for several aspects of visual development, albeit with varying definitions (Kiorpes 2015; Mitchell and Maurer 2022). At the neural level, such periods have been linked to inhibitory GABAergic circuitry, NMDA receptor activity, and neurotrophins (Hensch 2005; Berardi et al. 2000), as well as broader plasticity‐related processes, including synaptic pruning, axonal growth, and myelination (Huttenlocher 1979; Li et al. 2022; Nelson et al. 2008).

While the existence of such developmental periods is generally accepted, their time‐limited nature and potential functional significance warrant further investigation. On the one hand, a decline in plasticity over time may reflect intrinsic biological constraints or an energetically efficient trade‐off, given the metabolic costs of maintaining high neuronal plasticity. On the other hand, actively diminishing plasticity might itself have adaptive value. A key possibility examined in this paper is that a temporal decay of plasticity could help preserve the initial foundations of sensory mechanisms formed very early in development. Notably, this is the time period in which newborns are exposed to remarkably degraded sensory inputs, such as blurred (Dobson and Teller 1978) and color‐reduced (Adams and Courage 2002) imagery.

Building on past proposals (Turkewitz and Kenny 1982; Newport 1988; Elman 1993), the “adaptive initial degradation” hypothesis posits that early exposure to such degraded sensory inputs, rather than constituting a limitation of development, may actively help instantiate processing strategies that subserve robust perception later in life (L. Vogelsang, M. Vogelsang, Pipa, et al. 2024). In favor of this hypothesis, computational studies have demonstrated that networks initially trained on blurred images develop receptive fields tuned to lower spatial frequencies, subsequently improving generalization across blur levels in face classification (Vogelsang et al. 2018; Jang and Tong 2021) and, to a more limited extent, in object classification (Jang and Tong 2021; Avberšek et al. 2021; Yoshihara et al. 2023). In the color domain, early grayscale training was shown to yield robust luminance‐based representations, strongly enhancing resilience against later color variations for both face and object classification (M. Vogelsang, L. Vogelsang, Gupta, et al. 2024). Complementary behavioral work indicates that children who bypass such early degradations—especially those who gain sight at a more advanced developmental stage—often exhibit lasting perceptual deficits in tasks relying on such strategies (e.g., Geldart et al. 2002; Putzar et al. 2010; de Heering and Maurer 2014; McKyton et al. 2015; Vogelsang et al. 2018; M. Vogelsang, L. Vogelsang, Gupta, et al. 2024; Vogelsang, Gupta, et al. 2025). Collectively, these findings suggest that early sensory degradations may confer certain long‐term advantages.

Notably, in the computational studies described above, some of the benefits arising from initial training on degraded inputs do persist throughout subsequent training stages on full‐fidelity inputs. However, while this persistence appears fairly strong for the domain of color as well as for face classification in the acuity domain, it is markedly more limited for object classification in the context of visual acuity. Specifically, early‐established representations and classification strategies remain susceptible to interference or “catastrophic forgetting” when transitioning to later training stages with progressively clearer images (Jang and Tong 2021; Avberšek et al. 2021; Yoshihara et al. 2023). Consequently, training procedures employing mixed regimens, where inputs at varying blur levels are interleaved throughout training, have proven even more effective for robust object recognition (Avberšek et al. 2021; Yoshihara et al. 2023; Jang and Tong 2024). Thus, data augmentation approaches that randomize input fidelity can outperform strictly developmental training sequences. This observation motivates a key question of our current investigation: to what extent, across both acuity and color settings, do reductions in neuronal plasticity, modeled computationally as temporally‐decaying learning rates (LRs), help consolidate and strengthen early‐formed representations, thereby preserving more of their functional benefits into later developmental stages?

Motivated by this question, we employed convolutional neural networks to systematically manipulate both sensory input fidelity (degraded vs. non‐degraded) and plasticity (via different temporal LR trajectories) throughout training. Although deep networks have recognized limitations as direct proxies for biological vision (Wichmann and Geirhos 2023), they remain among the most powerful computational tools for predicting human‐like behavior and neural responses (e.g., Schrimpf et al. 2020; Lindsay 2021). Such models not only enable rigorous testing of neuroscientific hypotheses (Cichy and Kaiser 2019; Doerig et al. 2023) but also permit the systematic evaluation of developmental manipulations that would be ethically or practically infeasible in humans.

Specifically, we investigated here whether varying LR trajectories, including those that decrease over time as stand‐ins for time‐limited neuronal plasticity, might further enhance the benefits of commencing training with initially degraded inputs. As detailed below, we conducted comprehensive computational experiments in two separate domains—visual acuity (using blurred vs. clear images) and color vision (using grayscale vs. colored images). We examined both classification performance and the networks’ internal representations.

## 2 Methods

### 2.1 Computational Model

For the simulations presented in the main manuscript, we trained the AlexNet convolutional neural network (Krizhevsky et al. 2017) on the ImageNet object‐recognition database (Deng et al. 2009), which comprises 1000 distinct object classes. In brief, the AlexNet processes images through five consecutive convolutional layers followed by three fully‐connected layers. The early convolutional layers learn to detect simple visual features, such as edges and color gradients, with learned filters exhibiting some resemblance to basic receptive fields in the early visual cortex, while deeper layers tend to capture progressively more complex structures. Ultimately, the outputs of the last convolutional layer, which encode high‐level feature representations, feed into the fully‐connected layers to generate a final classification decision among the 1000 possible object categories.

During training, the randomly initiated filter shapes and connections in the final classification layers are iteratively refined through error minimization. Specifically, labeled images are presented to the network, and weights (i.e., filter parameters and classification‐related connections) are updated to reduce classification errors. Once trained, the learned filters and the model's resulting classification performance on the ImageNet test set can be examined to probe how the network represents and processes visual inputs.

**FIGURE 1:**
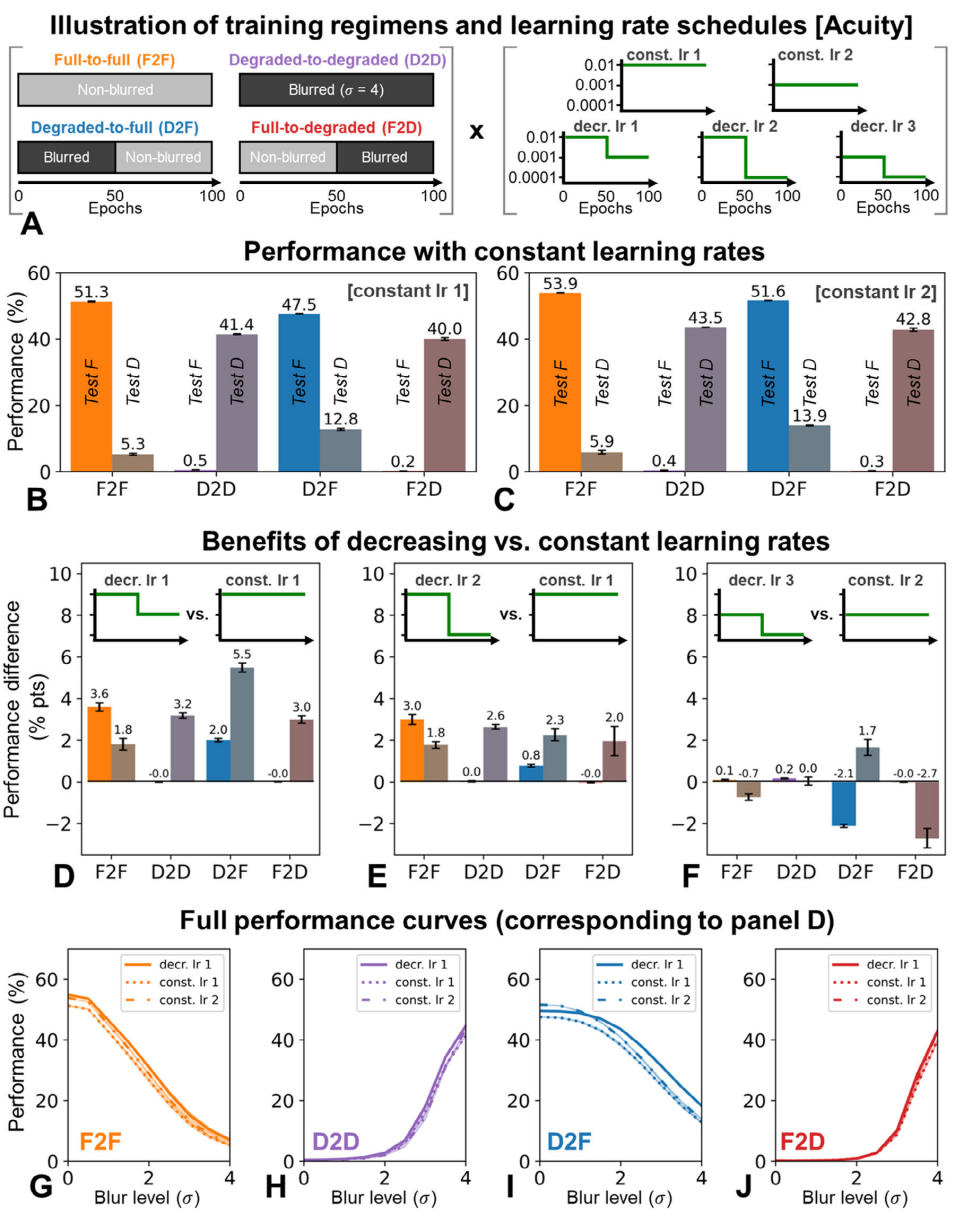
(A) Illustration of the different training regimens and learning rate schedules used. (B)–(C). Performance of our four networks trained with constant learning rates of 0.01 (B) and 0.001 (C) when tested on non‐blurred (full, "F") and blurred (degraded, "D") images. Depicted are means and standard errors across three training runs with different random initializations. (D)–(F). Performance benefits of using decreasing learning rates, when compared with corresponding constant learning rates (matched for initial value). Depicted are means and standard errors across training runs. (G)–(J). Classification performance as a function of test blur when trained with a constant learning rate of 0.01 or 0.001 versus when the learning rate decreased from 0.01 to 0.001. Mean values are shown as lines; shaded regions represent ±1 standard error.

**FIGURE 2:**
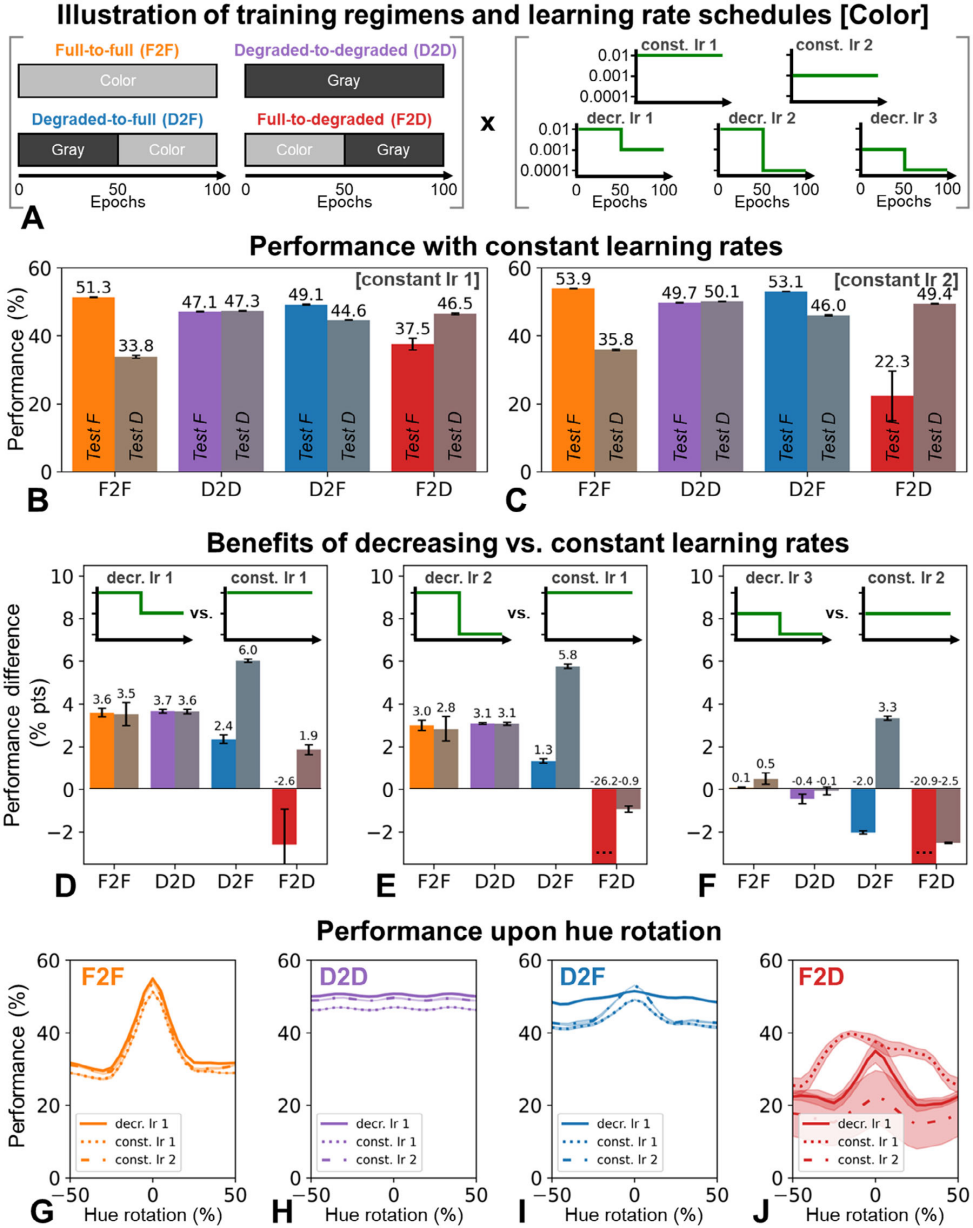
(A) Illustration of the different training regimens and learning rates used. (B)–(C) Performance of our four networks trained with constant learning rates of 0.01 (B) and 0.001 (C) when tested on color (full, "F") and grayscale (degraded, "D") images. Depicted are means and standard errors across three training runs with different random initializations. (D)–(F). Performance benefits of decreasing learning rate trajectories when compared with corresponding constant learning rates (matched for initial value). Depicted are means and standard errors across training runs. (G)–(J). Classification performance when test images are gradually hue‐rotated, following training with a constant learning rate of 0.01 or 0.001 versus when the learning rate decreased from 0.01 to 0.001. Mean values are shown as lines; shaded regions represent ±1 standard error.

To assess the stability of our key results, we carried out training of all models depicted in Figures 1, 2 with three random seeds and report means and standard errors across training runs. Further, to examine generalizability, we also replicated the AlexNet‐derived key findings reported in the main manuscript with the ResNet architecture (He et al. 2016). These results are presented in Figures S1–S2.

### 2.2 Network Training

We implemented and trained AlexNet using Keras/TensorFlow v2, adopting the official ImageNet split comprising more than one million training images across 1000 object classes, along with a test set of 50,000 images (50 per class). Training lasted for a total of 100 epochs and minimized categorical cross‐entropy loss via stochastic gradient descent (SGD) with a Nesterov momentum of 0.9 and a batch size of 128. Except for the manipulations described in Section 2.3, image preprocessing and data augmentation were kept fairly restricted and included only random 227 × 227 cropping from the 256 × 256 original images, pixel‐value rescaling from a range of [0, 255] to [–1, 1], and random horizontal flips. For the ResNet architecture, the overall procedure was the same, except that training lasted for only 40 epochs and that random crops had a size of 224 × 224 instead of 227 × 227 pixels.

### 2.3 Different Training Regimens

We compared four qualitatively distinct training regimens, which differed in how degraded versus full‐fidelity inputs were presented over time. Of these four stimulus trajectories, the “degraded‐to‐full” regimen was designed to (loosely) mimic the transition from degraded to undegraded inputs characteristic of early human development, with the other three serving as non‐developmental controls. Degradations involved either blurring the images with a Gaussian sigma (σ) of 4 (for experiments related to acuity) or converting them to grayscale (for color‐related experiments). Our full set of training regimens, each run for a total of 100 epochs, includes:

- Full‐to‐Full (F2F): Training comprised only clear, full‐color images for all 100 epochs.
- Degraded‐to‐Degraded (D2D): Training was carried out exclusively on degraded images (blurred or grayscale).
- Degraded‐to‐Full (D2F): This developmentally‐inspired regimen comprised training that begins with 50 epochs of degraded images and is followed by 50 epochs of full‐fidelity inputs.
- Full‐to‐Degraded (F2D): As the inverse of D2F, this regimen commenced with full‐fidelity inputs for 50 epochs and, for the final 50 epochs, switched to degraded inputs.

**FIGURE 3:**
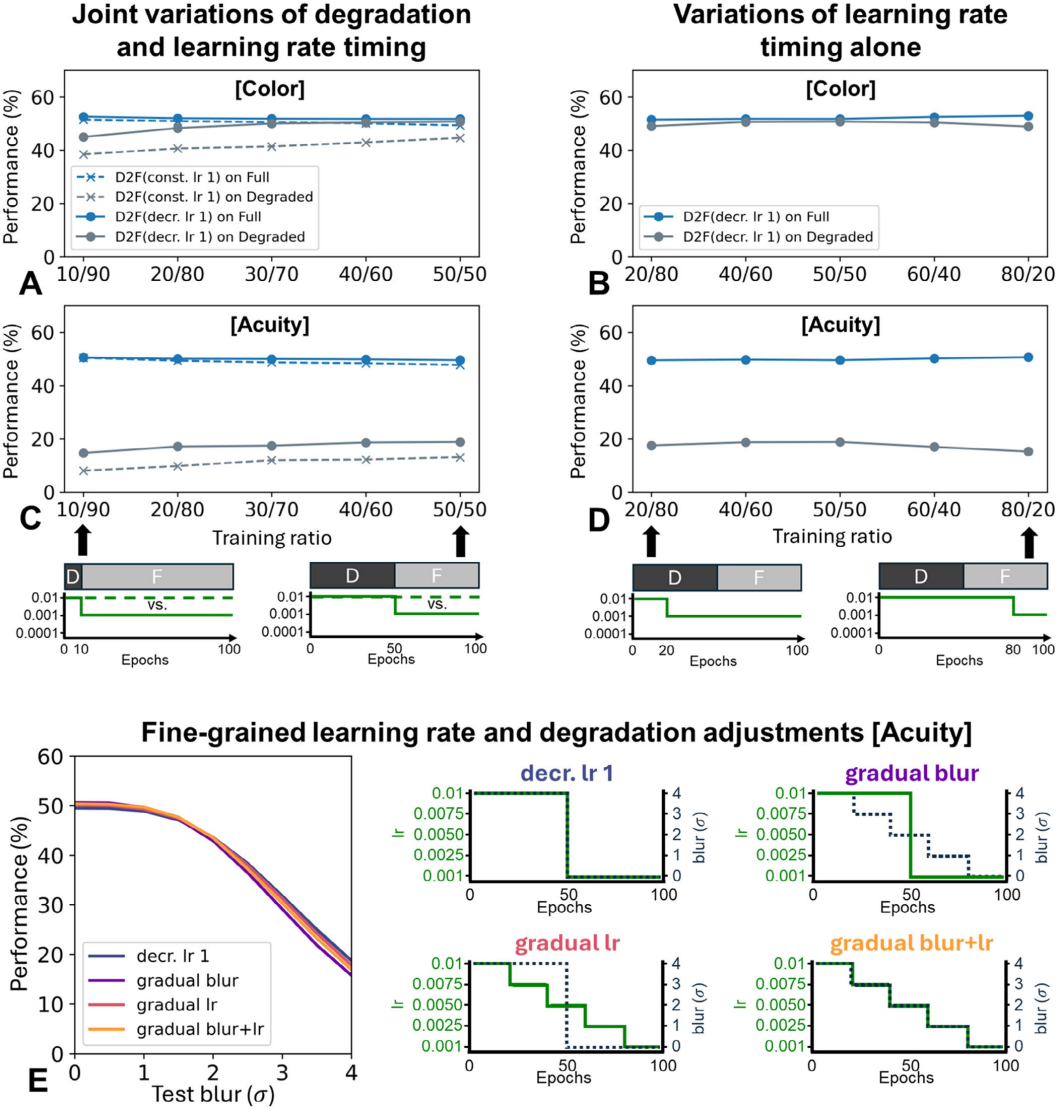
(A) and (C) Performance on full and degraded test images of networks trained with a constant learning rate of 0.01 (constant LR 1) and a learning rate decreasing from 0.01 to 0.001 (decreasing LR 1), as a function of the initial proportion of training on degraded inputs and high learning rates, separately for the domains of color (A) and acuity (C). (B) and (D) Performance on full and degraded test images when the training proportion of degraded/full images always comprises 50/50 epochs, but when the switch from high to low learning rates is varied in time, separately for color (B) and acuity (D). (E) Performance as a function of test blur for four different D2F models employing decreasing learning rates, featuring abrupt decay of both blur and learning rates, gradual decay of blur but abrupt decay of learning rates, abrupt decay of blur but gradual decay of learning rates, and gradual decay of blur and learning rates.

Additional manipulations, as reported in Figure 3, explored variations within the D2F regimen, such as training on degraded inputs for fewer than 50 epochs (specifically, for 10, 20, 30, or 40 epochs) and transitioning gradually from high blur to low blur (training, in this order, on blur levels 4, 3, 2, 1, and 0, for 20 epochs each).

### 2.4 Different LR Schedules

Alongside these training regimens, we systematically varied LR trajectories. Specifically, we employed three constant LRs and three schedules in which the LR decreased midway through training (after epoch 50). The complete set thus comprises:

- Constant LR 1: The LR was maintained at 0.01 throughout.
- Constant LR 2: The LR was maintained at 0.001 throughout.
- Constant LR 3: The LR was maintained at 0.0001 throughout.
- Decreasing LR 1: The LR started at 0.01 and dropped to 0.001 after epoch 50.
- Decreasing LR 2: The LR started at 0.01 and dropped to 0.0001 after epoch 50.
- Decreasing LR 3: The LR started at 0.001 and dropped to 0.0001 after epoch 50.

As shown in Figure 3, we also varied the timing of the LR drop, either simultaneously with, or independently from, the switch from degraded to full‐fidelity inputs (Figure 3A–D). In Figure 3E, we also feature a gradual LR decrease (from 0.01 to 0.0075, 0.005, 0.0025, and finally 0.001; every 20 epochs). Additionally, the consequences of using a common data‐driven reduce‐on‐plateau schedule (with initial LR = 0.01, factor = 0.5, patience = 5, minimum delta = 0, cooldown = 0, and minimum LR = 0), where LRs are reduced when reaching a plateau, are reported in Figure S3.

For direct comparisons between constant and decreasing LR schedules, each decreasing LR condition was compared against the corresponding constant LR that shared the same initial value. Thus, decreasing LR 1 (0.01 to 0.001) and decreasing LR 2 (0.01 to 0.0001) were compared against constant LR 1 (0.01), while decreasing LR 3 (0.001 to 0.0001) was compared against constant LR 2 (0.001). Comparisons matched for final LR (decreasing LR 1 compared to constant LR 2, and decreasing LRs 2 and 3 compared to constant LR 3) are shown in Figure S4.

### 2.5 Analyses

**FIGURE 4:**
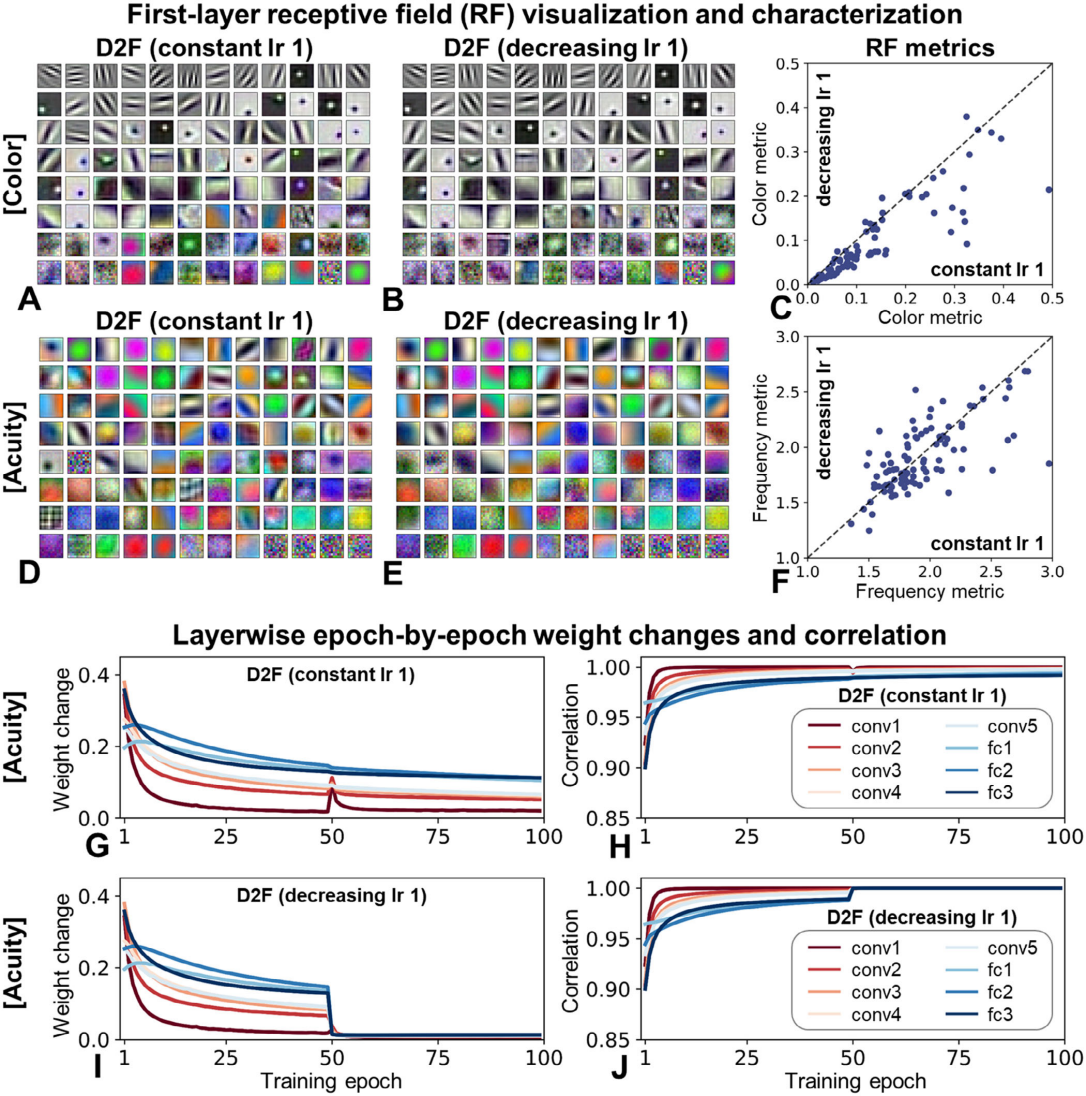
(A)–(B) and (D)–(E) First‐layer receptive field visualization for D2F models following training with a constant learning rate of 0.01 (constant LR 1) (A, D) versus with a learning rate decreasing from 0.01 to 0.001 (decreasing LR 1) (B, E), for simulations pertaining to color (A, B) and acuity (D, E). (C) and (F) Characterization of receptive field tuning to color (C) and spatial frequency (F) content. Depicted are individual RF metric scores for the D2F model trained with decreasing (left) and constant (bottom) learning rates. Values below the diagonal indicate receptive fields being tuned more to degraded stimulus characteristics when decreasing learning rates are employed. (G) and (I) Epoch‐by‐epoch weight changes as a function of training epoch and network layer. (H) and (J) Correlation coefficients between weights at a given epoch and weights at the subsequent epoch, as a function of epoch and network layer.

For each combination of training regimen and LR schedule, we evaluated model performance on both full‐fidelity and degraded images (blurred or grayscale) using the ImageNet test set. We additionally quantified receptive field (RF) tuning for color and spatial frequency content (Figure 4C–F) to assess how early training influenced later representations. We also tracked weight changes over the course of training to assess layer‐specific dynamics (Figure 4G–J). Complete details of these analyses are available in the Supplementary Methods.

### 2.6 Code and Data Availability

The code/data supporting the findings of this study are openly available at https://github.com/marin‐oz/TimeLimitedPlasticity.

## 3 Results

We trained AlexNet on four distinct training regimens, including a developmentally‐inspired D2F stimulus progression, across multiple LR trajectories, examining effects separately in the domains of visual acuity (using blurred vs. clear images) and color vision (using grayscale vs. colored images). Beginning with acuity, we first examined any benefits of the developmentally‐inspired regimen before moving on to specifically probing interaction effects with LR reductions. Next, we present analogous results for color vision. Finally, we explore the effects of timing and gradual training trajectories on observed performance, concluding with analyses of learned representations.

### 3.1 Benefits of Developmental Training Progressions in Visual Acuity

To establish a baseline, we first verified whether results obtained with constant LRs were consistent with previous findings highlighting the advantages (albeit incomplete ones) of developmentally‐inspired training progressions transitioning from blurred to clear images. Figure 1B,C depicts results for a constant LR of 0.01 (constant LR 1) and 0.001 (constant LR 2), respectively. Consistent across both, networks trained exclusively on clear images (F2F) performed well on clear images but generalized poorly to blurred ones. Conversely, networks trained only on blurred images (degraded‐to‐degraded, D2D) exhibited the opposite pattern. Importantly, networks trained with the developmentally‐inspired regimen transitioning from blurred to clear images (D2F), generalized best overall, though, akin to past studies, they still fell far short of perfect generalization and were likely inferior to mixed training procedures. Notably, the inverse‐developmental regimen (F2D) offered no such advantage, highlighting the role of the temporal ordering of input progressions and replicating the partial benefits of adaptive initial degradations in the acuity context. Similar, or even more pronounced, results emerge from simulations carried out with the ResNet architecture (Figure S1).

### 3.2 Modest Benefits Conferred by Temporally Decreasing LRs

Next, we investigated whether reducing LRs throughout training would further enhance generalization in the developmental D2F condition, hypothesizing it might solidify useful representations learned early on. Figure 1D–F depicts comparisons between results obtained with decreasing and constant LRs, when matched for the initial LR. Specifically, Figure 1D compares an LR reduction from 0.01 to 0.001 halfway through training (decreasing LR 1) to a constant LR of 0.01 throughout. Similarly, Figure 1E compares a reduction from 0.01 to 0.0001 (decreasing LR 2) against a fixed rate of 0.01, and Figure 1F compares a reduction from 0.001 to 0.0001 (decreasing LR 3) against a fixed rate of 0.001.

Consistent with our predictions, across all comparisons, the D2F model benefited from decreasing LRs, showing improved performance primarily for blurred images (the type presented during the first half of training). Interestingly, however, the inverse‐developmental F2D model also showed slight performance improvements for blurred images. Given that, for the F2D model, these images are presented in the second half of training, the slight benefits observed are not specifically attesting to the preservation of early‐acquired representations. Similarly, uniform training regimens (F2F and D2D) showed some modest general performance improvements when employing decreasing LRs, suggestive of general optimization‐related improvements upon LR reduction.

Overall, the benefits of decreasing LRs observed for the D2F networks when classifying degraded images point to possible advantages of time‐limited neuronal plasticity in preserving early‐established mechanisms. These results were stable across random training runs and also persisted consistently across blur levels (Figure 1G–J), underscoring their robustness. Moreover, the advantages of decreasing over constant LRs are also evident when matched for the final LR (Figure S4), indicating that the results are not due to a comparison with a too‐high constant LR. Nevertheless, in absolute terms, effects remained surprisingly modest (only up to 6 percentage points) and were even more modest and less specific when replicated with the ResNet architecture (Figure S1). Additionally, the benefits observed for other training regimens suggest at least partial contributions from general network optimization advantages conferred by decreasing LRs.

### 3.3 Similarly, Modest Benefits of LR Reductions Emerge in the Color Domain

Akin to the case of visual acuity, fairly similar results emerge for the domain of color vision. Figure 2B,C demonstrates superior generalization of the D2F regimen (initially grayscale, then color) compared to F2F and F2D regimens, consistent with M. Vogelsang, L. Vogelsang, Gupta, et al. (2024) and more pronounced than in Figure 1. Moreover, unlike in the case of acuity, networks trained exclusively on degraded (grayscale) inputs (D2D) generalized well, aligning with previous findings that highlight specific advantages of D2F over D2D training for certain image subsets and visual tasks outside of the domain of recognition (M. Vogelsang, L. Vogelsang, Gupta, et al. 2024).

Figure 2D–F depicts the effects of decreasing versus constant LRs. Across all tested settings, the D2F model consistently, albeit modestly, exhibited benefits for performance on degraded (grayscale) inputs, more so than other regimens. Figure 2G–J also demonstrates these benefits when images undergo systematic variations of hues, again revealing specific gains for the D2F model. These findings were stable across training runs, with the exception of the F2D network, which appears to exhibit greater instability and thus varies markedly more substantially across training runs. The results also hold for comparisons matched for final LR (Figure S4) and were generally replicated with the ResNet architecture, although revealing even more modest and less specific effects (Figure S2).

Thus, analogous to the findings presented in the context of acuity (Figure 1), the results reveal systematic but only small benefits of decreasing LRs in developmentally‐inspired training. These modest improvements might reflect both the stabilization of early‐learned representations and general optimization‐based benefits of LR decreases.

### 3.4 Finer‐Grained Simulations Reveal Limited Significance of Precise Timing and Gradual Training Trajectories

We conducted three further analyses exploring developmentally relevant and timing‐related factors to test whether they underlie some of the consistent but modest effects described above.

First, we examined whether shorter initially‐degraded training periods prior to LR reductions would magnify the benefits of decreasing LRs (decreasing LR 1) over constant ones (constant LR 1). This is motivated by the observation that sensory maturation observed in biological development can be fairly rapid (Kiorpes 2015). However, contrary to this expectation, Figure 3A,C shows no clear relationship for either color or acuity; instead, the benefits of decreasing LRs remained relatively constant regardless of initial training proportions.

Second, we varied only the timing at which the LR is reduced from 0.01 to 0.001 (decreasing LR 1), while maintaining equal training proportions on degraded and undegraded inputs (each featuring 50 epochs). As is evident from Figure 3B,D, we again found no notable differences. These results suggest generally low sensitivity of our simulations to precise timing.

Third, acknowledging that real development involves gradual reductions in sensory degradation and neural plasticity, we compared abrupt versus gradual training changes in the acuity domain. Specifically, in addition to an abrupt blur and LR reduction after 50 epochs (decreasing LR 1), we tested gradual blur reductions with an abrupt LR drop (gradual blur), gradual LR reductions with an abrupt blur change (gradual LR), and simultaneous gradual reductions in both (gradual blur + LR). Across these conditions, only minimal differences are evident (Figure 3E). This suggests that the simpler discrete transitions we used previously appear to adequately approximate developmental dynamics relevant to the current investigation.

Finally, it is worth noting that a common adaptive LR schedule triggered by performance plateaus (reduce LR on plateau) also yielded similar results, as depicted in Figure S3.

### 3.5 Analyses of Learned Representations and Weight Changes Across Layers Reveal Potential Parallels With the Biological System

As the effects reported above could potentially relate to the stabilization of processing mechanisms acquired early in training, we next examined the models' representational underpinnings. Previous studies demonstrated that blurred training biases receptive fields toward lower spatial frequencies, with grayscale training inducing biases toward reduced color tuning. These biases partially, but not perfectly, persisted through later clear/color training (Vogelsang et al. 2018; M. Vogelsang, L. Vogelsang, Gupta, et al. 2024). Motivated by this observation, we tested whether decreasing LRs during later training phases might further enhance the retention of these early biases at the representational level.

For the domain of color, Figure 4 depicts receptive fields from D2F models trained with constant (Figure 4A) versus decreasing (Figure 4B) LRs. Figure 4C quantifies the tuning of individual receptive fields to color content, revealing that receptive fields indeed remained more grayscale‐biased under decreasing LRs. This is indicated by most points falling below the diagonal (86 out of 96). A similar but weaker effect was observed for spatial frequency tuning in the case of visual acuity (Figure 4D–F; 57 out of 96 being below the diagonal).

This investigation also prompted a more general consideration of layer‐specific representational dynamics during training. Examining epoch‐to‐epoch weight changes in the D2F network (see Supplementary Methods for details) revealed relatively fast representational stabilization during training, even if trained with a constant LR (Figure 4G). This is even more evident when weight changes are quantified in terms of correlations between subsequent epochs, thereby disregarding any changes in, for instance, the absolute strength of filters (Figure 4H). Unsurprisingly, stabilization is particularly evident upon LR decrease (Figure 4G–J).

A notable finding in this regard is that early network layers generally appear to stabilize sooner than later layers. This may account for the rather modest first‐layer changes observed in Figure 4A–F. It also aligns with biological findings of early visual layers similarly reducing plasticity first—a point we revisit in the Discussion section. While Figure 4G–J is constructed on the basis of our acuity‐related simulations, corresponding results based on our color simulations are included as Figure S5.

## 4 Discussion

Across both acuity and color domains, our findings reveal surprisingly subtle but consistent benefits of reducing LRs during training, particularly in developmentally inspired models transitioning from training on degraded inputs to full‐fidelity images later. In these models, improvements primarily emerge for the degraded stimulus domain (i.e., blurred or grayscale images), suggesting that processing mechanisms established early in development could potentially become stabilized through a subsequent reduction in “plasticity,” here approximated by lowered LRs. Additional support for this interpretation derives from our analyses of first‐layer receptive fields, which, following training with decaying LRs, remain slightly more tuned to characteristics of degraded inputs, especially in the color domain. Nonetheless, the overall magnitude of these effects is remarkably modest, indicating circumscribed impact of plasticity reduction over time. This finding appears generally consistent with past results on training with progressively clearer object images, reported separately for constant LRs (Jang and Tong 2021) and decreasing ones (Avberšek et al. 2021; Yoshihara et al. 2023).

Moreover, learning‐rate decay also confers benefits for networks that lack an initial degradation phase, hinting at more general optimization advantages. These observations are consistent with computationally‐observed benefits of employing decaying LRs and align with past proposals that larger initial LRs help avoid overfitting to noise, while subsequent rate reductions refine more complex patterns and improve convergence (You et al. 2019). Thus, part of the advantage we see in developmentally‐inspired regimens might reflect some of these general optimization gains, alongside the stabilization of early degradation‐based representations as the LR is reduced.

A secondary finding we obtained is that the color domain exhibits somewhat more pronounced benefits than the acuity domain for both developmental stimulus progressions and decreasing LRs. One possibility is that, in the context of object classification, generalization across blur levels may be more demanding than generalization across hues. Past computational work in the domain of visual acuity has also suggested class‐dependent outcomes, with blurred‐to‐clear regimens yielding substantial improvements for face recognition but very modest effects for the recognition of other categories of objects (Jang and Tong 2021).

Third, our layer‐specific analyses indicate that earlier network layers tend to stabilize sooner than deeper layers, loosely mirroring evidence that lower‐level visual functions become less malleable at younger ages, whereas higher‐level functions remain plastic longer (Mitchell and Maurer 2022; Sinha and Held 2012). Similarly, processes like synaptic pruning and myelination typically conclude earlier in lower sensory areas than in higher cognitive regions (Tierney and Nelson 2009; Nelson et al. 2008). While it remains unclear whether these parallels reflect similar underlying mechanisms, the resemblance is at least suggestive of potential functional parallels between maturing neural circuits and deep network trajectories.

From a broader perspective, our work contributes to the discussion of the stability‐plasticity dilemma, which focuses on how learning systems maintain existing knowledge and avoid catastrophic interference while continually learning new tasks (e.g., Kim and Han 2023; Jung et al. 2023; Chen et al. 2023). Although our simulations do not involve multi‐task learning, the principle of progressively reducing plasticity to consolidate prior learning remains relevant to that debate.

Turning to the adaptive value of developmental degradations, our findings reinforce earlier arguments that initial exposure to degraded inputs can instantiate useful feature representations and improve generalization (Turkewitz and Kenny 1982; Newport 1988; Elman 1993; Jinsi et al. 2023; Cheon and Paik 2025; Lu et al. 2025; Vogelsang et al. 2018; Vogelsang et al. 2023; L. Vogelsang, M. Vogelsang, Pipa, et al. 2024; M. Vogelsang, L. Vogelsang, Gupta, et al. 2024; M. Vogelsang, L. Vogelsang, Pipa, et al. 2025), although the exact benefits can vary, and mixed degraded regimens, compared to strict developmental sequences, may confer additional advantages (Jang and Tong 2021; Avberšek et al. 2021; Yoshihara et al. 2023). It is important to acknowledge that the payoffs for reducing plasticity appear smaller in the contexts we tested than those conferred by sensory degradations themselves.

While our simulations help isolate the interaction between early sensory degradations and declining plasticity (modeled here by reductions in LR), several limitations deserve emphasis, each presenting interesting opportunities for future research.

First, although deep neural networks effectively model certain aspects of human vision, they still diverge significantly from biological vision both behaviorally (Wichmann and Geirhos 2023; Bowers et al. 2023) and in terms of neural connectivity (Kreiman and Serre 2020). For example, our strictly feedforward architecture lacks recurrent connections, which benefit complex visual processing (Tang et al. 2018) and more accurately reflect human neural dynamics (Kietzmann et al. 2019). Incorporating biologically‐inspired connectivity patterns, including any developmental trajectory differences between feedforward and feedback circuits (Berezovskii et al. 2011), could offer further developmental insights. Future studies could systematically test whether architectures incorporating these constraints replicate or even amplify the developmental advantages observed here.

Second, our approximation of declining neuronal plasticity through decreasing LRs, while intuitive, remains a highly simplified representation of complex biological processes, including inhibitory circuitry, myelination, and widespread connectivity changes (Hensch 2005; Nelson et al. 2008). Historically, diverse computational modeling methods have been used to explicitly simulate developmental plasticity (see Yermolayeva and Rakison 2014, for review). For instance, constructive algorithms, such as cascade‐correlation (Fahlman and Lebiere 1989), dynamically add hidden units during training, thereby modeling circuit recruitment beyond mere weight adjustments, and have successfully captured aspects of cognitive development (Shultz 2003). Additionally, approaches combining modeled synaptogenesis with synaptic pruning, such as Mayor and Plunkett's (2010) use of self‐organizing maps with initially exuberant connectivity followed by selective elimination, have effectively modeled early learning processes. Inspired by these methodologies, future investigations into time‐limited plasticity in deep networks could incorporate explicit mechanisms for adding or eliminating connections as well as selectively freezing weights in a time‐dependent manner. Such modifications could also be applied for each layer separately, as inspired by past biological findings (Tierney and Nelson 2009; Nelson et al. 2008). Notably, Delrocq et al. (2024) recently demonstrated that enforcing strict, layer‐staggered critical periods in a biologically plausible generalized Hebbian learning framework with a predictive component improves the quality and stability of sensory representations. In such a context, it would be interesting to test whether stronger interactions with stimulus fidelity could potentially be observed.

Third, our simulations intentionally focused on isolated sensory degradations (either color or acuity) and employed a fixed object‐classification task (ImageNet). However, real‐world human development involves concurrent maturation across multiple sensory modalities and ecologically richer tasks. Recent computational studies of simultaneous degradations across different sensory dimensions have provided additional developmental insights into aspects such as global representation learning, perturbation robustness, and neural pathway organization (Cheon and Paik 2025; Lu et al. 2025; M. Vogelsang, L. Vogelsang, Pipa, et al. 2025). Moreover, training models on more naturalistic datasets, such as egocentric baby‐cam recordings, has emerged as a particularly powerful strategy for learning high‐level representations (Orhan and Lake 2024). Future studies could build on these approaches, extend simulations to continuous or sequential learning paradigms, and possibly incorporate the time domain, which may critically scaffold perceptual development (Sinha et al. 2025). This would not only enhance ecological validity but could also uncover additional developmental effects. Furthermore, examining higher‐order cognitive functions beyond basic object recognition might reveal even stronger or qualitatively different advantages conferred by initial sensory degradations and time‐limited plasticity.

Addressing the above‐stated open questions offers pathways toward achieving deeper computational insights into developmental neuroscience. Additionally, conditions associated with hyperplasticity, such as autism, highlight the functional significance of time‐limited plasticity windows for typical neural organization (Wilson et al. 2017; Oberman and Pascual‐Leone 2014). Future investigations could thus also hold potentially broader significance for atypical development.

Notwithstanding these open questions, our results underscore the value of incorporating developmentally driven constraints, both in terms of early degraded inputs and time‐limited plasticity, into models of sensory learning. They also extend support for the hypothesis that developmental degradations offer certain advantages by showing that even a mild, time‐limited reduction in plasticity may modestly amplify those benefits. Such enhancements also illustrate the emerging synergy between advancing developmental science and machine learning (Zaadnoordijk et al. 2022).

## Ethics Statement

No human data were collected in this study.

## Conflicts of Interest

The authors declare no conflicts of interest.

## Supporting information




**Supporting File 1**: desc70099‐sup‐0001‐SupMat.docx

## Data Availability

The code/data supporting the findings of this study are openly available at https://github.com/marin‐oz/TimeLimitedPlasticity.
