# Supplementary material for: Computationally Probing the Role of Time‐Limited Neuronal Plasticity in Early Visual Development
Source: Dev Sci. 2025 Nov 22;29(1):e70099. doi: 10.1111/desc.70099 (PMC12640114; doi:10.1111/desc.70099)
Supplement: Supplementary file 1 — Supporting File 1: desc70099‐sup‐0001‐SupMat.docx [file DESC-29-e70099-s001.docx]

**Supplementary Material**

The supplementary material accompanying the main manuscript ‘Computationally probing the role of time-limited neuronal plasticity in early visual development’ contains:

Supplementary Figures 1-5.

Supplementary Methods.


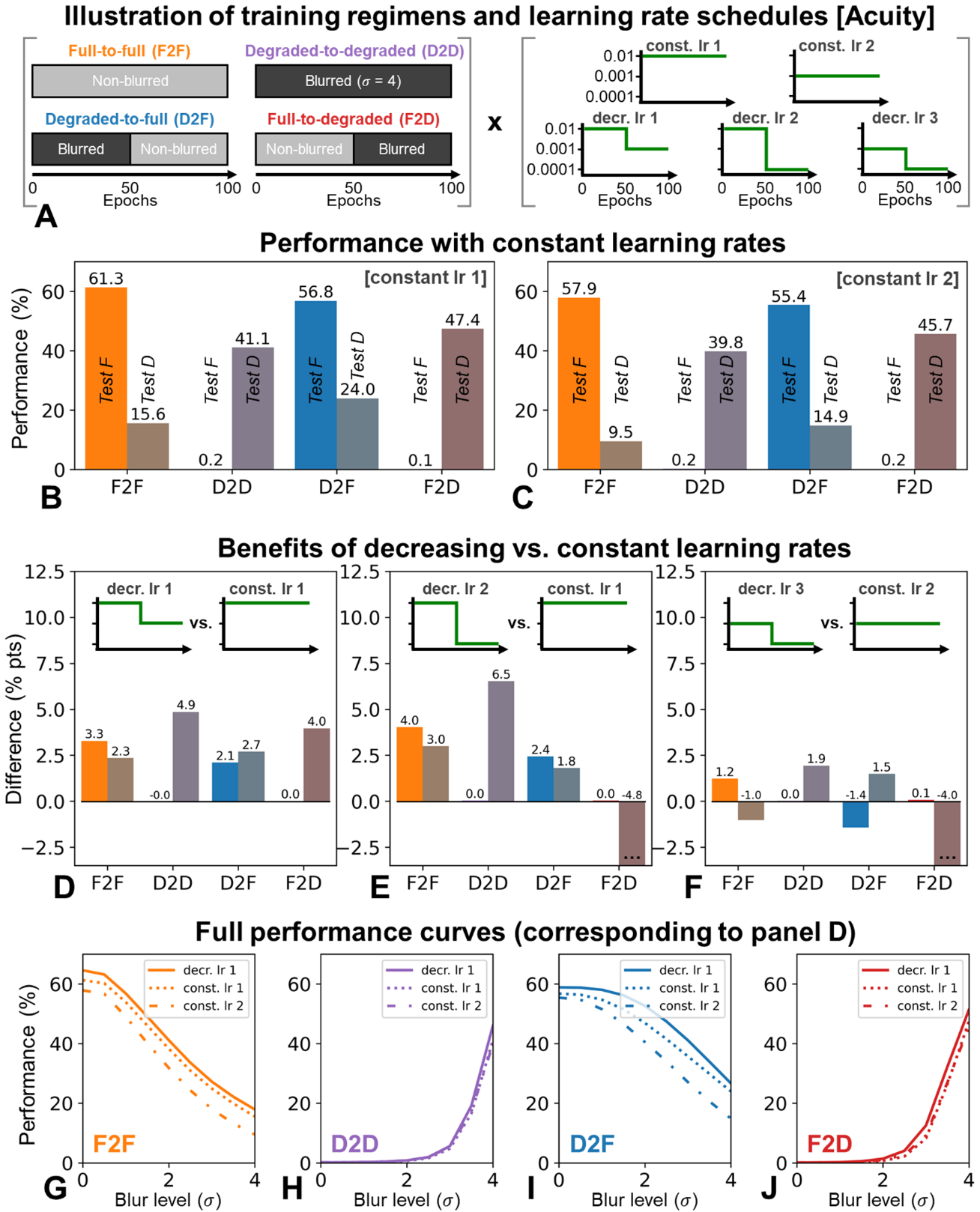


***Supplementary Figure 1.*** *Replication of the main results presented in Figure 1 using the ResNet-50.* ***A.*** *Illustration of the different training regimens and learning rate schedules used.* ***B-C.*** *Performance of our four networks trained with constant learning rates of 0.01 (B) and 0.001 (C) when tested on non-blurred (Full, ‘F’) and blurred (Degraded, ‘D’) images.* ***D-F.*** *Performance benefits of using decreasing learning rates, when compared with corresponding constant learning rates (matched for initial value).* ***G-J.*** *Classification performance as a function of test blur when trained with a constant learning rate of 0.01 or 0.001 vs. when the learning rate decreased from 0.01 to 0.001.*


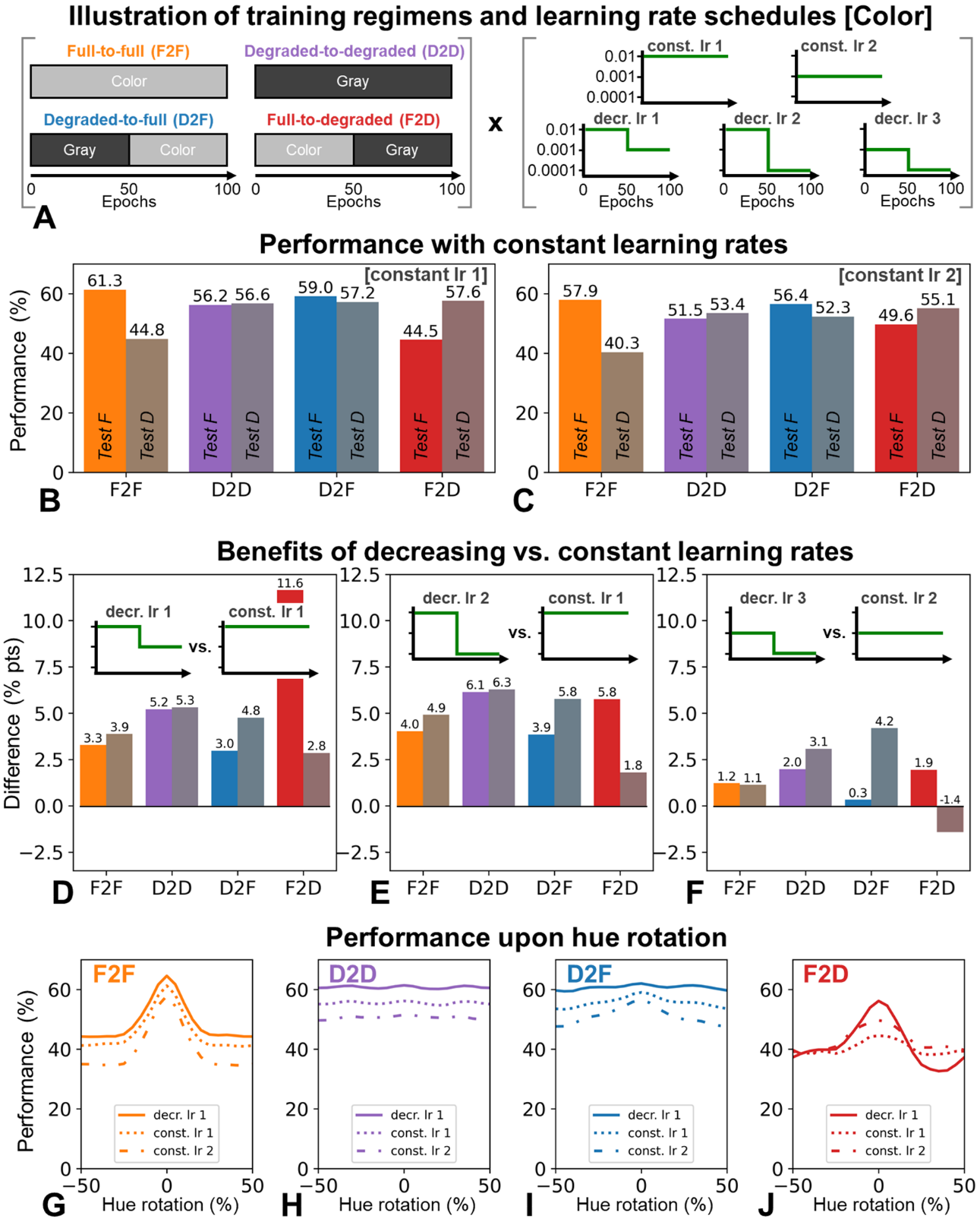


***Supplementary Figure 2.*** *Replication of the main results presented in Figure 2 with the ResNet-50.* ***A.*** *Illustration of the different training regimens and learning rates used.* ***B-C.*** *Performance of our four networks trained with constant learning rates of 0.01 (B) and 0.001 (C) when tested on color (Full, ‘F’) and grayscale (Degraded, ‘D’) images.* ***D-F.*** *Performance benefits of decreasing learning rate trajectories when compared with corresponding constant learning rates (matched for initial value).* ***G-J.*** *Classification performance when test images are gradually hue-rotated, following training with a constant learning rate of 0.01 or 0.001 vs. when the learning rate decreased from 0.01 to 0.001.*


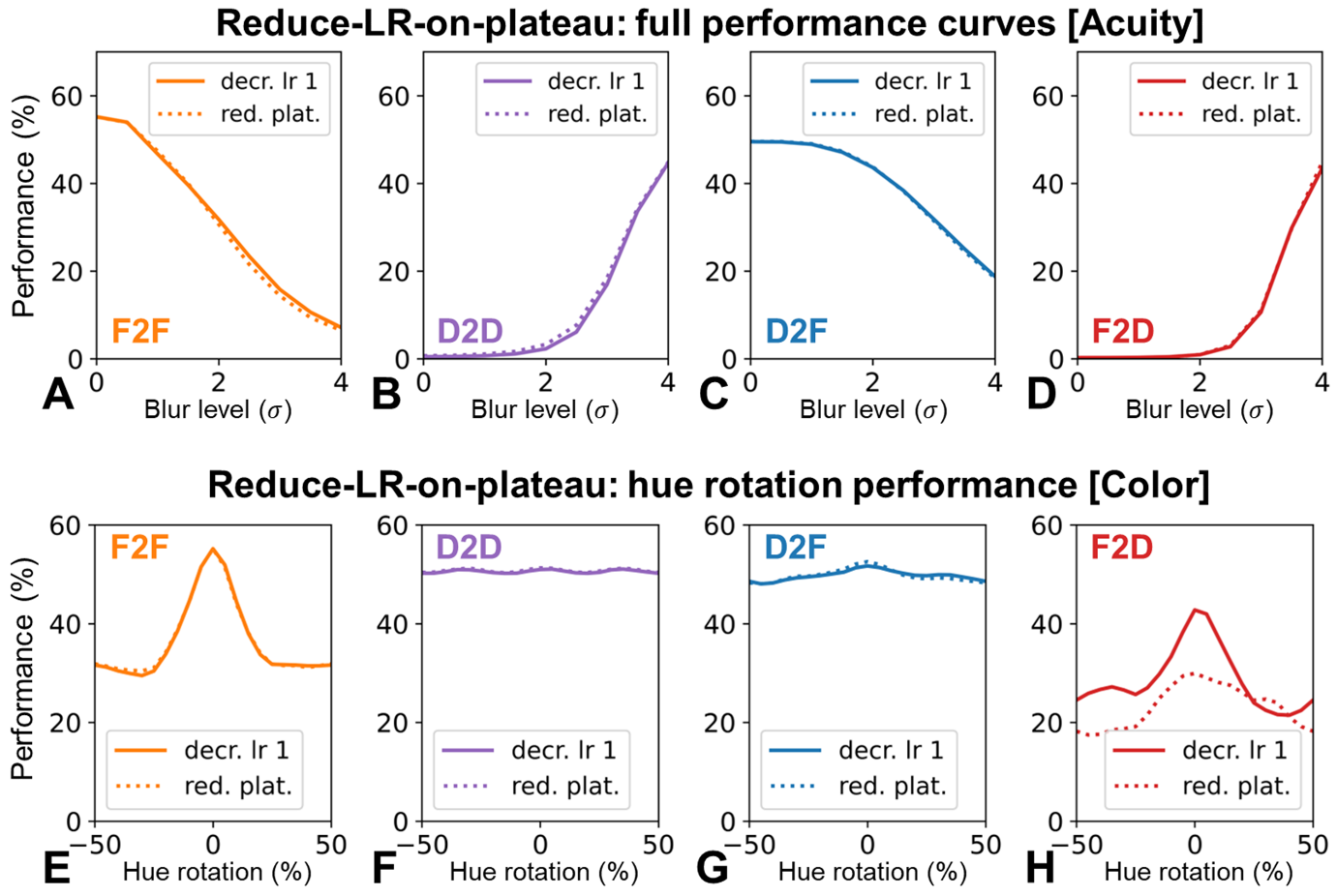


***Supplementary Figure 3.*** *Comparison between a learning rate decreasing from 0.01 to 0.001 vs. when following a reduce-on-plateau procedure (see Methods for details).* ***A-D.*** *Classification performance as a function of test blur, for simulations in the acuity domain.* ***E-H.*** *Classification performance when test images are gradually hue-rotated, for simulations in the color domain.*

*
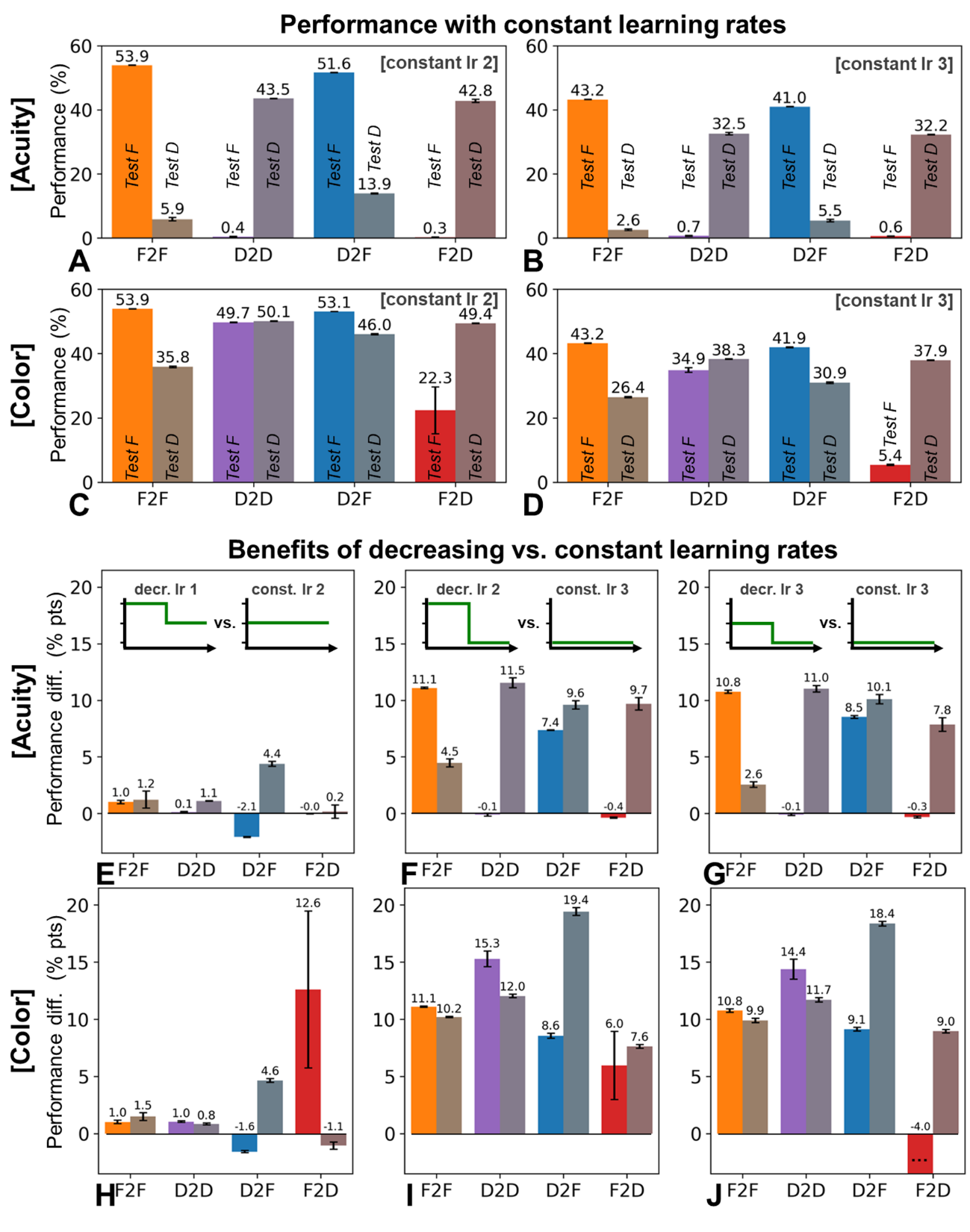
*

***Supplementary Figure 4.*** *Replication of Figures 1B-F & 2B-F when matching decreasing and constant learning rates with regard to the final, not initial, value.* ***A-D.*** *Performance of our four networks trained with constant learning rates of 0.001 (A, C) and 0.0001 (B, D), in the acuity (A-B) and color domain (C-D). Depicted are means and standard errors across three training runs with different random initializations.* ***E-J.*** *Performance benefits of using decreasing learning rates, when compared with constant learning rates matched for the final learning rate, separately for the acuity (E-G) and color (H-J) domain. Depicted are means and standard errors across training runs.*


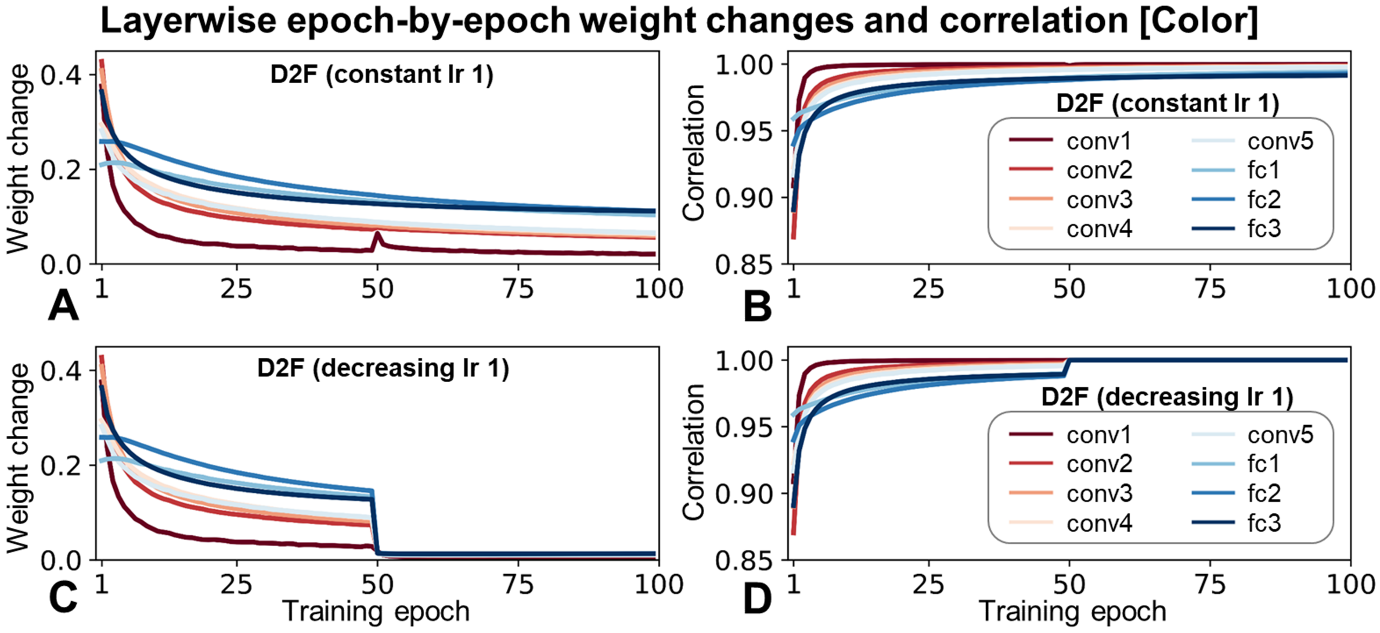


***Supplementary Figure 5.*** *Replication of Figures 4G-J for the color domain.* ***A&C.*** *Epoch-by-epoch weight changes as a function of training epoch and network layer.* ***B&D.*** *Correlation coefficients between weights at a given epoch and weights at the subsequent epoch, as a function of epoch and network layer.*

**Supplementary Methods**

**Color RF metric (Figure 4C)**

We quantified each RF’s color sensitivity in two steps. First, for each pixel in a given RF, we extracted the discrepancy of individual color channels, *m*, across the R, G, and B channels, as follows:

$$x=R\cos0^{\circ}+G\cos120^{\circ}+B\cos-120^{\circ}$$

$$y=R\sin0^{\circ}+G\sin120^{\circ}+B\sin-120^{\circ}$$

$$m=\sqrt{x^{2}+y^{2}}$$

R, G, and B thereby represent pixel-wise channel intensities. To determine each filter’s overall color sensitivity, we then computed the mean of *m* across all pixels in the 11x11 RF.

**Spatial frequency RF metric (Figure 4F)**

To capture the spatial frequency content of each first-layer RF, we converted it to grayscale and applied a 2D Fast Fourier Transform (2D-FFT). We then performed radial averaging to obtain a 1D amplitude vector as a function of frequency and computed the weighted average frequency as follows:

$$weighted average frequency= \frac{\sum_{f} amp\left( f \right)*f}{\sum_{f} amp\left( f \right)}$$

where *amp* refers to the amplitude of a given frequency *f*. Note that the constant part of the FFT was excluded. Further, to mitigate any discrete indexing artifacts, each 11x11 RF was up-sampled by a factor of 100 prior to application of the 2D-FFT.

**Quantification of weight changes and correlation (Figure 4G-J):**

For each of the 100 training epochs and each of the 8 layers in the AlexNet, we extracted the network weights and flattened them into 1D vectors, resulting in 800 vectors for each model instance. To quantify changes between consecutive epochs, we computed the absolute difference between the flattened weight vectors for adjacent epochs and summed these values across weights. We then normalized by dividing by the sum of the absolute values of the later epoch’s weight vector, thereby yielding a relative measure of per-epoch weight updates.

Similarly, using the flattened 1D weights for each layer at each epoch, we computed the correlation coefficient between weights at consecutive epochs. This provided a scale-invariant measure of how similar a layer’s weights remained as training progressed from one epoch to the next.
